# Modulation of anti-tumour immunity by XPO1 inhibitors

**DOI:** 10.37349/etat.2025.1002310

**Published:** 2025-04-23

**Authors:** Jack G. Fisher, Laura G. Bartlett, Trinayan Kashyap, Christopher J. Walker, Salim I. Khakoo, Matthew D. Blunt

**Affiliations:** University of Salford, UK; ^1^Clinical and Experimental Sciences, University of Southampton, SO16 7YD Southampton, UK; ^2^Karyopharm Therapeutics, Newton, MA 02459, USA

**Keywords:** Exportin-1 (XPO1), selinexor, immunotherapy, CAR-T, CAR-NK, ADCC

## Abstract

Exportin-1 (XPO1) is a nuclear export protein that, when overexpressed, can facilitate cancer cell proliferation and survival and is frequently overexpressed or mutated in cancer patients. As such, selective inhibitors of XPO1 (XPO1i) function have been developed to inhibit cancer cell proliferation and induce apoptosis. This review outlines the evidence for the immunomodulatory properties of XPO1 inhibition and discusses the potential for combining and sequencing XPO1i with immunotherapy to improve the treatment of patients with cancer. Selinexor is a first-in-class XPO1i that is FDA-approved for the treatment of patients with relapsed and refractory (RR) multiple myeloma and RR diffuse large B cell lymphoma. In addition to the cancer cell intrinsic pro-apoptotic activity, increasing evidence suggests that XPO1 inhibition has immunomodulatory properties. In this review, we describe how XPO1i can lead to a skewing of macrophage polarisation, inhibition of neutrophil extracellular traps, modulation of immune checkpoint expression, blockade of myeloid-derived suppressor cells (MDSCs) and sensitisation of cancer cells to T cell and NK (natural killer) cell immunosurveillance. As such, there is an opportunity for selinexor to enhance immunotherapy efficacy and thus a need for clinical trials assessing selinexor in combination with immunotherapies such as immune checkpoint inhibitors, direct targeting monoclonal antibodies, chimeric antigen receptor (CAR)-T cells and cereblon E3 ligase modulators (CELMoDs).

## Introduction

Gene expression is a highly regulated biological process controlled at multiple levels, from chromatin accessibility, gene transcription, and RNA modification to protein translation. One of these mechanisms is the spatial separation of signaling molecules, transcription factors, RNA and ribosome constituents. Transport of this material between the nucleus and cytoplasm is governed by karyopherins, a GTPase protein family containing importins, exportins and biportins [1]. Karyopherins recognise unique motifs within cargo to initiate cargo import/export between the nucleus and cytoplasm. For example, exportin-1 (XPO1, also called *CRM1*) recognises a leucine-rich nuclear export signal (NES) sequence to translocate cargo proteins to the cytoplasm [1].

During oncogenesis, gene expression becomes dysregulated to induce the hallmarks of cancer, such as sustained replication and resistance to apoptosis [2]. XPO1 can augment tumourigenesis through virtually all of the hallmarks of cancer, including evasion of immune surveillance [3]. XPO1-mediated nuclear export impacts multiple cellular oncogenic mechanisms. One of these is the translocation of tumour suppressor transcription factor proteins and DNA damage repair proteins such as p53 and BRCA1 out of the nucleus, resulting in their physical separation from DNA and de facto inhibition, protecting cancer cells from apoptosis [4–8]. Secondly, with the help of adapter proteins, XPO1 can export oncogenic mRNA species from the nucleus, where they are transcribed, to the cytoplasm and translated into oncoproteins. These include oncogenes involved in cell division and cell cycle progression, such as *MYC*, retinoblastoma protein (*RB*) and cyclin D1 (*CCND1*) [9, 10]. Thirdly, XPO1 traffics ribosome constituents and rRNA to impact global protein translation rates, allowing for sufficient protein production to support sustained cell proliferation [11–13].

As a consequence of XPO1 function facilitating cell proliferation and resistance to apoptosis, the overexpression of XPO1 [14] and mutations enabling stronger interactions with cargoes and altering the XPO1 interactome [15, 16] have been observed in many solid and haematological cancers. Not surprisingly, high expression of XPO1 is correlated with poor clinical outcomes across a range of cancer types [17]. Because tumours depend on the function and expression levels of XPO1 for survival and proliferation, selective inhibitors of XPO1 have been developed. The first clinically utilized XPO1 inhibitor (XPO1i) is the anti-tumour *Streptomyces* antibiotic leptomycin B (LMB), which is a natural XPO1i that covalently attaches to the cargo binding pocket of XPO1 to potently inhibit its interaction with cargo proteins [18]. When cancer cells are treated with LMB, cell proliferation is impaired in addition to the induction of apoptosis [19]. However, phase I clinical trials for patients with refractory cancer were halted due to severe toxicities and no observations of response [20]. Semi-synthetic LMB derivatives demonstrate comparable XPO1 inhibition to LMB with much improved in vivo toxicity [21]. Following this, a plethora of synthetic XPO1i compounds were developed with potent anti-tumour effects but with tolerable/manageable in vivo toxicity due to the reversible nature of XPO1 inhibition [14, 22].

The FDA has approved the first-in-class XPO1i selinexor (KPT-330) for the treatment of relapsed and refractory (RR) multiple myeloma and RR diffuse large B cell lymphoma (DLBCL). In addition, there are multiple ongoing clinical trials involving selinexor for haematological and solid cancers in combination with therapies that promote anti-cancer immunity (Table 1). Notably, many of the listed trials are early phase and/or investigator initiated, with smaller sample sizes, and some were terminated due to enrollment concerns. Therefore, additional clinical investigation, either based on real-world evidence or larger clinical trials are warranted to validate the findings. To maximise the efficacy of selinexor in combination with other therapies, it is essential to determine the consequence of XPO1 inhibition beyond cancer cell apoptosis and impaired cell proliferation. XPO1 interacts with hundreds of cargoes [23, 24], therefore, its inhibition potentially modulates a multitude of biological processes. For example, XPO1i may impact the immunogenicity of cancer cells, which may provide an approach to tailoring selinexor-immunotherapy combinations to promote more robust anti-cancer responses in patients.

**Table 1:** Clinical trials assessing the combination of selinexor with immunotherapy

**Cancers**	**Indication**	**Immunotherapy combination with selinexor**	**NCT number**	Status*	**Phase**
Haematological cancers	Double hit & triple hit lymphoma	Rituximab + CHOP	NCT05974085	Recruiting	II
	RR B cell lymphoma	Rituximab + GDP or rituximab + DHAOx	NCT02741388	Completed	I
	RR B cell lymphoma	Rituximab + ICE	NCT02471911	Completed	I
	EBV+ DLBCL	Rituximab + CHOP	NCT05577364	Recruiting	I/II
	RR DLBCL & indolent NHL	Rituximab + lenalidomide	NCT05265975	Recruiting	I/II
	RR B-NHL	CD19-CAR-T	NCT05322330	Unknown	II
	RRDLBCL	Rituximab + GDP	NCT04442022	Recruiting	II/III
	GCB-DLBCL	Rituximab + CHOP	NCT05422066	Recruiting	II
	R/RCLL NHL	Ibrutinib	NCT02303392	Unknown	I
	RR multiple myeloma (MM)	Daratumumab, pomalidomide or carfilzomib + dexamethasone	NCT04661137	Recruiting	II
	Newly diagnosed (ND) MM	Lenalidomide + bortezomib + dexamethasone	NCT05422027	Recruiting	I/II
	RR MM	Lenalidomide, thalidomide, or ​pomalidomide​ + dexamethasone	NCT04941937	Recruiting	II
	RR MM	Pomalidomide + dexamethasone	NCT05028348	Recruiting	III
	RR MM	Mezigdomide + dexamethasone	NCT02343042	Recruiting	I/II
	RR MM	Daratumumab + bortezomib + dexamethasone	NCT03589222	Unknown	II
		Daratumumab + carfilzomib + dexamethosone	NCT04756401	Active, not recruiting	II
		Lenalidomide	NCT04519476	Recruiting	I
	RR MM	Daratumumab, pomalidomide or carfilzomib + dexamethasone	NCT04925193	Active, not recruiting	II
	ND MM	Lenalidomide or bortezomib + dexamethasone	NCT04717700	Active, not recruiting	II
	High risk, ND MM	Daratumumab + bortezomib + dexamethasone	NCT06169215	Recruiting	II
	ND MM with extramedullary disease	Lenalidomide + bortezomib + dexamethasone	NCT05900882	Recruiting	II
	RR extramedullary MM	BCMA-CAR-T	NCT05201118	Unknown	I
	MM & myeloma-associated amyloidosis	Lenalidomide + dexamethasone	NCT05820763	Withdrawn	II
	ND MM	Daratumumab + lenalidomide + dexamethasone	NCT04782687	Active, not recruiting	II
Solid cancers	Advanced solid malignancy (not including brain tumors)	Ipilimumab Nivolumab Pembrolizumab Additional chemotherapy arms	NCT02419495	Terminated	I
	NSCLC or CRC	Pembrolizumab FOLFIRI Docetaxel	NCT04256707	Recruiting	I/II
	ND HCC	Bevacizumab + atezolizumab	NCT05093608	Terminated	I
	Advanced/Metastatic solid malignancy	Nivolumab + ipilimumab	NCT04850755	Unknown	I
	Advanced/Metastatic urothelial carcinoma	Pembrolizumab	NCT04856189	Recruiting	I/II
	Alveolar soft part sarcoma	Atezolizumab	NCT05333458	Recruiting	II
	Recurrent advanced melanoma	Pembrolizumab	NCT04768881	Terminated	II

CAR: chimeric antigen receptor; DLBCL: diffuse large B cell lymphoma; RR: relapsed and refractory; BCMA: B cell maturation antigen; GCB: germinal center B-cell; NHL: non-Hodgkin lymphoma. *Trial status provided is according to information on clinicaltrials.gov as of (Date—01/23/2025); respective trial sponsor should be contacted for further clarification/information

Modulation of the immune system by anti-cancer agents beyond their intrinsic anti-proliferative and/or pro-apoptotic functions is becoming increasingly evident [25, 26]. For example, histone deacetylase (HDAC) inhibitors have shown wide-ranging immunomodulatory effects. Tumour regression induced by the pan-HDAC inhibitor panobinostat in mice is dependent on the presence of natural killer (NK) cells [27] and HDAC inhibitors improved the efficacy of anti-CD20-based therapeutics, including monoclonal antibodies, by enhancing the expression of CD20 [28, 29]. Dasatinib, an ABL tyrosine kinase inhibitor (TKI) used to treat chronic myeloid leukaemia (CML), can downregulate the inhibitory receptor NKG2A on patient NK cells, which promotes NK cell function against CML cells [30]. TKIs also suppress STAT signaling, disrupting the immunosuppressive effects of regulatory T cells (Tregs) and myeloid-derived suppressor cells (MDSCs) alongside modulating immune checkpoint receptor expression. As such, TKIs have shown synergy with dendritic cell-based immunisation in lymphoma mouse models [31]. Small molecule inhibitors targeting tyrosine phosphatases can promote T cell function whilst perturbing tumour growth signaling pathways [32]. The proteasome inhibitor bortezomib can downregulate HLA-E, the ligand for the inhibitory NKG2A receptor, and increase surface expression of death receptors on cancer cells to promote NK cell cytotoxicity against multiple myeloma cells [33]. Therefore, understanding the cellular processes effected by XPO1 inhibition will enable the design of tailored therapeutic combinations to improve efficacy. In this review, we discuss how XPO1 expression and its pharmacological inhibition modulate immune effector functions and explore the potential of combining XPO1i with immunotherapy.

## Expression of XPO1 & the implications on the immune system

Clinical studies in DLBCL have given insights into how XPO1 may affect cancer and the anti-cancer immune response. In a cohort of 4,665 DLBCL patients, *XPO1* overexpression was found to be overrepresented in the resistant/relapsed subgroup, and interestingly, *XPO1* expression was negatively correlated with IFNγ signaling, suggesting dysfunctional or reduced frequency of immune cells within the tumour microenvironment (TME) [8]. Similarly, a study that measured *XPO1* expression in DLBCL patient samples with different levels of immune infiltrates noted that immune deficiency was associated with high *XPO1* expression [34]. Oncogenic mutations of *XPO1* can also associate with a negatively impacted immune system, with a study of germinal center B-cell (GCB)-DLBCL patients showing that those with a gain-of-function mutation in XPO1 possessed lymphodepleted microenvironments [35]. Overall, these studies illustrate that elevated XPO1 expression and/or mutation-driven gain of function correlate with an immunosuppressive environment in DLBCL.

An in-depth investigation into *XPO1* expression in wide-ranging, solid and haematological cancer types found negative correlations between *XPO1* expression and immune scores as calculated using the ESTIMATE (estimation of stromal and immune cells in malignant tumour tissues) algorithm [36]. This included decreased activated NK cells and decreased CD8^+^ T cell abundance with increased expression of *XPO1*. Additionally, *XPO1* expression was positively correlated with immune checkpoint expression and negatively correlated with co-stimulatory receptors, suggesting that *XPO1* expression is associated with an immunosuppressive microenvironment [36]. In lung adenocarcinoma, amplification of *XPO1* was associated with decreased infiltration of B cells, CD4^+^ T cells and dendritic cells [37].

Recurrent codon E571 mutations within the *XPO1* cargo binding groove have been reported in multiple cancer types but most frequently occur in B-cell malignancies [38, 39]. These mutations impact cargo binding affinity to XPO1, resulting in altered gene expression, and are validated oncogenic mutations, as the E571 *XPO1* mutation has been shown to drive an accelerated rate of leukemogenesis in mouse models of chronic lymphocytic leukaemia (CLL) [40–42]. CLL patients with *XPO1* E571 mutations had poor prognosis and harboured altered IFNγ, granzyme B and CD28 expression, which may contribute to the overall immune dysfunction seen in CLL patients [40, 43].

Besides cancer, XPO1 function is also associated with impaired immune function during infection. For example, Kaposi sarcoma-associated herpesvirus (KSHV) relies on XPO1 function to enable effective replication within cells [44]. Upon XPO1 inhibition, immune-related genes were increased, which limited KSHV replication. Human cytomegalovirus (HCMV) replication was also impaired with XPO1i via nuclear retention of interferon regulatory factor 3 (IRF-3) and activation of interferon-stimulating genes [45]. This is context-dependent, however, as during pathological inflammation with influenza [46] or COVID-19 [47], XPO1 inhibition dampened immune activation, reducing levels of IFNγ, TNF and IL-6, among other cytokines, which can be attributed to the inhibition of hyperactivated pro-inflammatory pathways like NF-κB pathway [47].

Together, these studies highlight that XPO1 expression and/or mutation status is correlated with altered immune infiltrates and expression of immune-related genes across multiple cancer types, which may have implications for tumour escape in patients. In the sections below, we review the current literature on the effect of XPO1 inhibition on specific cells of the immune system involved in the anti-cancer response.

## The effect of XPO1 inhibition on myeloid cells

Macrophages are the most abundant cells within the TME and are highly plastic, differentiating either into M1-like anti-tumourigenic or M2-like pro-tumourigenic cells [48, 49]. During oncogenesis, the TME can promote M2-like polarisation of tissue-resident macrophages and M2-like differentiation of infiltrating monocytes via cytokines such as IL-4, CSF and the secretion of exosomes [50]. These cells then generate an immunosuppressive TME via the secretion of anti-inflammatory cytokines such as IL-10 and TGFβ which impede NK cell and T cell anti-tumour immunity [51]. As such, intense research is currently directed at better understanding the role of tumour resident macrophages and how to target them to reinstate effective anti-tumour immune responses. One strategy is to deplete M2-like macrophages, for example, by using chimeric antigen receptor (CAR)-T cells [52]. Another approach being evaluated is the use of small molecule inhibitors to facilitate the selective depletion of pro-tumourigenic macrophages [53]. Indeed, selinexor has recently been shown to selectively deplete lymphoma-associated macrophages (LAM) ex vivo and in vivo*,* resulting in impaired tumour growth and improved survival of mice [54].

An alternative macrophage-targeting approach is to alter the phenotype of M2-like macrophages into anti-tumourigenic, immunostimulatory M1-like macrophages, and there is emerging evidence showing that XPO1 inhibition can re-polarise macrophages in the TME. In mouse models of primary central nervous system lymphoma, selinexor shifted macrophage polarisation from M2-like to M1-like within the TME [55]. When combined with ibrutinib, this shift to M1 remained, whilst ibrutinib monotherapy did not affect polarisation [55]. Thus, the effects of XPO1i may not be impacted by Bruton’s tyrosine kinase (BTK) inhibitors, indicating that this combination forms a rational anti-cancer strategy given the evidence of overlapping impact on key tumorigenic pathways like p53, mTOR and NF-κB. In addition to re-polarisation, M2 macrophages showed decreased expression of programmed cell death protein 1 (PD-1) and SIRPα, a checkpoint receptor that inhibits phagocytosis after selinexor treatment [55]. Furthermore, CSF-1R, a cytokine involved in M2-like differentiation that is being targeted by novel anti-cancer therapies [56], has been reported to decrease LAM and is depleted after selinexor treatment [54]. Collectively, these data indicate that XPO1i may affect the phenotype of tumour-associated M2-like macrophages and selectively deplete these cells from the TME, promoting an anti-tumour immune response, in addition to the direct cytotoxic activity of XPO1i against cancer cells.

In contrast, selinexor in combination with gemcitabine-nab-paclitaxel (Sel‐GemPac) has also been shown to increase macrophage abundance within the TME in murine models of pancreatic cancer using immunohistochemistry staining of the mouse macrophage marker F4/80 [57]. F4/80 staining is not exclusive to M1- or M2- macrophage, so additional studies are required to further understand the increase in immune response post-Sel-GemPac treatment. However, with the longer overall survival for Sel‐GemPac treated mice, one would suspect tumor suppressive M1-macrophages to be more prevalent in tumour tissues. In melanoma-bearing mice, selinexor increased infiltration of MDSC, a group of cells consisting of neutrophils and monocytes which suppress immune functions within the TME [58]. It is worth noting that in the same model selinexor slowed tumor growth, so whether MDSCs are limiting tumour regression in this scenario remains to be resolved. It remains unclear why macrophage depletion occurs in specific tumour settings with selinexor and not in others, and clarification of this is required in future studies. In a separate study, selinexor was shown to convert human MDSCs from immunosuppressive cells to immunostimulatory cells with anti-tumor functions and blunt the immunosuppressive effects of MDSC in a mouse model of lymphoma [59]. Recently, LILRB1 and LILRB2 receptors, which are highly expressed on macrophages and transduce inhibitory signals, have been shown to interact with HLA-E [60]. We have demonstrated previously that HLA-E is downregulated with XPO1i [61, 62] and, by potentially reducing inhibitory LILRB1/2 signaling, this may have a positive effect on macrophage function including increased phagocytic capabilities and increased cytokine production, although this remains to be determined.

In patients treated with selinexor, neutropenia can be a significant side effect in addition to other common side effects like nausea and thromobocytopenia [63]. However, in addition to reduced neutrophil number, selinexor has been shown to impair extracellular trap formation by neutrophils [64]. In cancer, neutrophils are recruited to the TME and are activated to release extracellular traps, which promote tumour growth and metastasis [65], and thus may represent another means by which selinexor modulates immune-driven responses in the TME.

The effect of XPO1i on myeloid cells seems to be context dependent. For instance, selinexor can reduce pro-inflammatory signaling in mouse models of sepsis by reducing serum levels of pro-inflammatory cytokines IL-6 and TNF-α and also reducing macrophage numbers in the peritoneal cavity [66]. As such, selinexor can improve the survival of mice with LPS-induced sepsis. In vitro, LPS incubation of human peripheral blood mononuclear cells (PBMC) with selinexor showed impaired production of inflammatory cytokines, including IFNγ and IL-1β [47]. Likewise, the combination of selinexor and ruxolitinib in patients with myelofibrosis is associated with decreased pro-inflammatory cytokines [67]. The proinflammatory cytokine reductions were validated in a study of single-agent selinexor for patients with ruxolitinib refractory or intolerant myelofibrosis [68]. However, IL-10 production, an anti-inflammatory cytokine, was also reduced with selinexor. It must be noted that downregulation of IL-10 can be beneficial in the middle phase of infections as it triggers a rapid amplification of the innate and adaptive immune response and facilitates effective clearance of invading pathogens [69]. In sum, the collective effect on the immune system requires further investigation. These studies suggest that XPO1 inhibition may help to prevent cytokine storms during viral infections and circumvent adverse immune-related events, but whether this is true for cancer remains to be resolved [70].

## The effect of XPO1 inhibition on NK cell function

NK cells are key mediators of anti-tumour immunity and play essential roles in direct tumour lysis and regulating adaptive immune responses via the secretion of cytokines and chemokines [71]. NK cells lyse tumour targets via multiple mechanisms, including secretion of cytotoxic granules containing perforin and granzymes, engagement of death receptors and antibody-dependent cellular cytotoxicity (ADCC).

In murine models of melanoma, splenic NK cell abundance increased with XPO1 inhibition, and numbers in the TME were unchanged compared to vehicle controls [58, 72]. These studies did not assess NK cell function within these tissues, however, an in vitro study showed that NK cell ADCC against malignant B cells was unaffected by XPO1i [73].

**Figure 1:**
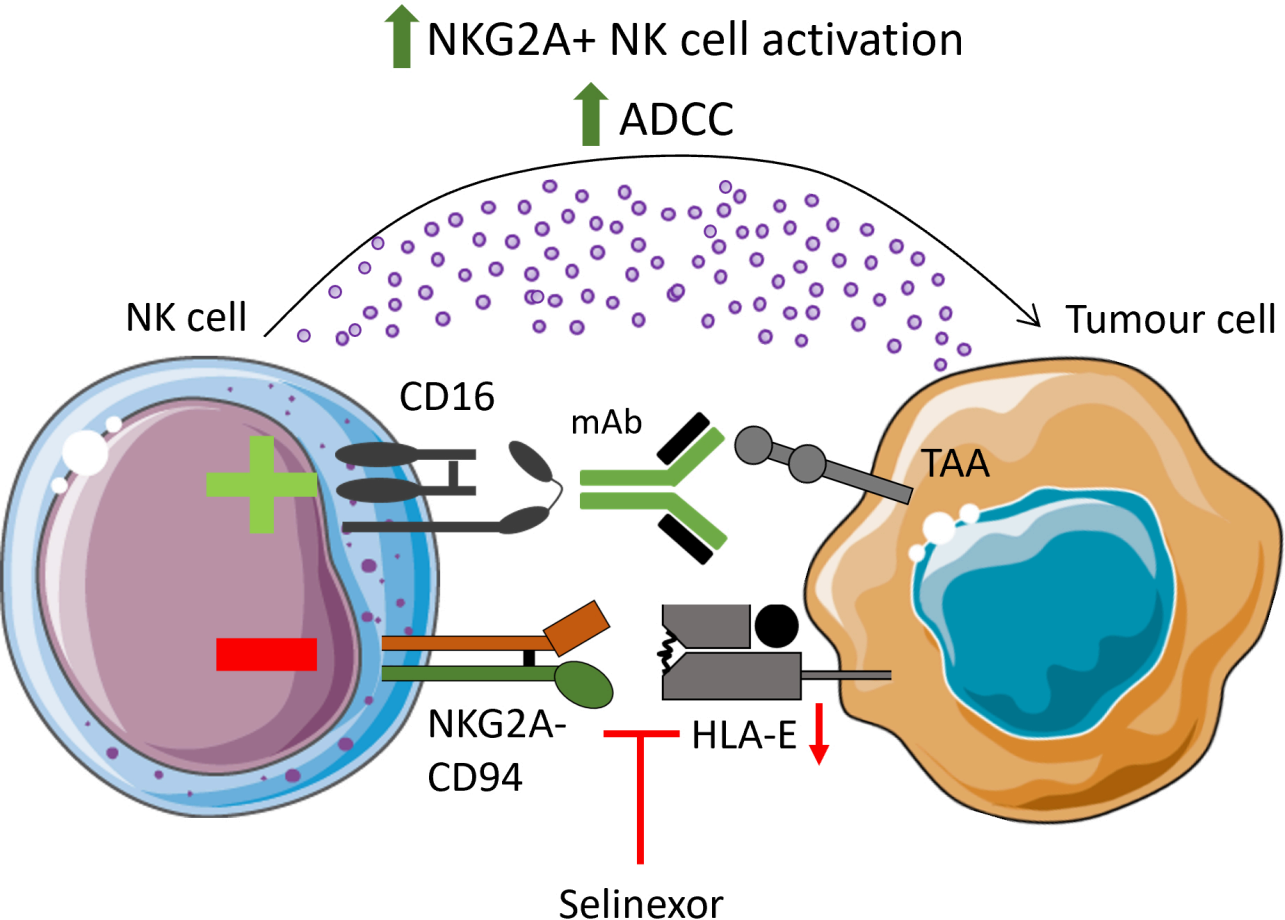
**Selinexor enhances NK cell function**. Treatment of malignant B cells with selinexor has been shown to enhance NK cytotoxicity via downregulation of HLA-E, decreasing interactions with the inhibitory NK cell receptor NKG2A. When combined with clinically relevant monoclonal antibodies targeting tumour-associated antigens (TAAs), selinexor enhanced ADCC of opsonised targets. ADCC: antibody-dependent cellular cytotoxicity; NK: natural killer; mAb: monoclonal antibody. Parts of this figure were adapted from pictures provided by Servier Medical Art (https://smart.servier.com/) under a Creative Commons Attribution 4.0 Unported License (https://creativecommons.org/licenses/by/4.0/)

Our research on NK cells has illustrated that pre-treatment of B-cell lymphoma cells and primary CLL cells with selinexor promotes NK cell-mediated anti-tumour immunity [61, 62]. The mechanism behind this was revealed to be selinexor-mediated downregulation of HLA-E on malignant B cells, leading to reduced inhibitory signaling by NKG2A on NK cells (Figure 1). NKG2A is a key inhibitory receptor for NK cells, and HLA-E is highly expressed on a wide range of tumours compared to healthy cells [74, 75]. As such, the NKG2A:HLA-E inhibitory axis is a significant pathway exploited by tumours to evade NK cell-mediated immunity. Strategies to overcome NKG2A signaling are undergoing intense research, exemplified by a phase 3 clinical trial with the monoclonal antibody monalizumab for lung cancer [76].

The lymph nodes are a key tissue site for drug resistance and tumour cell survival in CLL, and interestingly, selinexor overcomes high HLA-E expression induced by the lymph node-derived signals IL-4 and CD40L to enhance NK activation against CLL [62]. Given that HLA-E is heavily influenced by cytokines and other signals [77, 78], it will be important to dissect the immunogenic properties of XPO1 inhibition in other models of human cancers which recapitulate the TME in patients.

Furthermore, alongside downregulation of HLA-E, selinexor can enhance death receptor surface expression on CLL cells, which can improve NK-mediated lysis via engagement with TNF-related apoptosis-inducing ligand (TRAIL) [62]. Therefore, two parallel mechanisms for improved NK cell function with XPO1i have been demonstrated: one that reduces negative NKG2A-mediated signaling and one that enhances death receptor signaling.

Through expression of CD16, NK cells contribute to tumour-targeting monoclonal antibody (mAb) therapy [79]. Anti-CD20 mAbs are first-line treatments for CLL and non-Hodgkin lymphoma. Combination of selinexor with the clinically relevant anti-CD20 monoclonal antibodies rituximab and obinutuzumab further enhanced NKG2A^+^ NK cell activation [61, 62]. This also held true for the anti-CD38 mAb daratumumab, demonstrating that enhanced ADCC in the presence of selinexor is not antigen-specific (Figure 1). These data emphasize the potential of combining selinexor with NK-stimulating immunotherapies, and indeed, selinexor is currently being assessed in multiple clinical trials in combination with ADCC-inducing antibodies, including rituximab, daratumumab and elotuzumab (Table 1).

Selinexor underwent clinical testing with the BTK inhibitor ibrutinib for CLL and non-Hodgkin lymphoma (NCT02303392). However, ibrutinib is known to impair NK cell function via off-target inhibition of ITK [80] and so when selinexor and ibrutinib were added to cancer-NK cell co-cultures, ibrutinib reversed the immune-stimulating effect of selinexor [62]. However, NK cell function was partially restored when selinexor was combined with the more selective BTK inhibitor acalabrutinib, which is less disruptive to NK cell function. This emphasizes that to harness selinexor’s immune-stimulating function, it is crucial to understand how drug combinations impact immune cell function.

NK cells are currently in clinical trials as an allogeneic cell therapy [71], whereby expanded and activated NK cells or CAR-NK cells are adoptively transferred into patients. As an anti-cancer therapy, allogeneic NK cells have shown promising efficacy in hematological malignancies with an improved safety profile compared to T cell therapies, with no evidence of GvHD nor cytokine release syndrome [81]. Interestingly, during NK cell expansion, NKG2A is significantly upregulated [82, 83], and high HLA-E expression on multiple myeloma cells can limit CAR-NK cell function, which can be restored with NKG2A blockade [84]. Moreover, NKG2A blockade can promote expanded NK cell cytotoxicity against HLA-E^+^ cancer cells [82, 85]. As such, because selinexor is known to downregulate surface HLA-E on malignant B cells and can further enhance NKG2A^+^ NK cell function, it will be interesting to determine whether XPO1 inhibition can potentiate the activity of CAR-NK cells against B cell malignancies.

## The effect of XPO1 inhibition on T lymphocytes

Approved immune checkpoint inhibitors (ICIs) that target T lymphocytes can promote their priming in secondary lymphoid organs and/or their cytotoxic function at the tumour site. The targets of ICIs approved for cancer treatment are PD-1/programmed death ligand 1 (PD-L1), cytotoxic T-lymphocyte associated protein 4 (CTLA-4), and lymphocyte activation gene-3 (LAG-3), and XPO1 inhibition has been shown to modulate the expression of these molecules. Although a study reported that selinexor increased *PD-L1* and *CTLA-4* transcript expression in mouse melanoma cells [58], we saw no evidence for increased PD-1 or CTLA-4 expression on human melanoma cells following treatment in a phase 2 clinical trial (NCT04768881) assessing selinexor in combination with pembrolizumab (unpublished observations). In addition, assessment of paired pre- and post-selinexor-treated samples from patients with multiple myeloma revealed no induction of immune checkpoints on CD138^+^ myeloma cells [86]. In these patients, the CD3^+^ T-cells showed increased granzyme B, especially for those who received the selinexor, pomalidomide, and dexamethasone combination, consistent with selinexor-inducing T-cell activity. Similarly, murine studies of selinexor showed reduced LAG-3, T-cell immunoglobulin and mucin-domain containing-3 (TIM-3) and PD-1 expression on tumour-infiltrating CD8^+^ T cells [72, 87]. Preclinical models have demonstrated that XPO1i reduces T cell exhaustion, and this is thought to be due to a combination of factors, including the facilitation of a favorable microenvironment, reduced expression of immune checkpoints and activation of cytoprotective transcription factors [87]. Additionally, selinexor’s impact on MDSC and macrophages can influence microenvironmental signals which can regulate T cell exhaustion, for example, XPO1 blockade transforms MDSCs into T-cell-activating neutrophil-like cells [59].

Treatment of B16 melanoma-bearing mice with selinexor alone can also change the systemic immune landscape [58] and in the spleen, CD4^+^ and CD8^+^ T cell activation was enhanced as measured by CD62L and CD44 positivity. Interestingly, immune checkpoints on CD8^+^ T cells were downregulated with selinexor at the tumour site, and although not significant, granzyme B and IFNγ expression were increased, highlighting an active immune response within the TME with selinexor [72]. In contrast, selinexor decreased splenic T cell secretion of pro-inflammatory cytokines, including IFNγ in ovarian cancer mouse models [88]. This highlights the importance of understanding the characteristics of XPO1 inhibition in different cancer models and organs and the short-versus long-term benefits of pro-inflammatory signaling on T cell immune surveillance.

The dosing schedule of selinexor is critical to consider when combining selinexor with immunotherapy agents, as this can impact the anti-tumour immune response considerably. Tyler et al. [72] (2017) demonstrated optimal immune function when mice were administered selinexor twice weekly compared to thrice weekly. With optimal twice-weekly dosing, CD8^+^ T cell tumour infiltrates showed markers of enhanced activation along with decreased expression of immune checkpoints and unaltered Treg infiltration. Thrice weekly selinexor disrupted normal immune homeostasis, reducing T cell abundance in the bone marrow and thymus and impairing B cell development. In vitro, T cell receptor (TCR) signaling and T cell proliferation were impaired with high concentrations of selinexor and the expression of the activation markers CD25, CD69, IFNγ and granzyme B were reduced. It is important to note that the twice-weekly dosing schedule did not reveal any significant differences in the populations of FoxP3 positive Tregs [72]. Clinically, most of the investigational studies have adopted once-weekly dosing (Table 1), and selinexor once weekly is approved with bortezomib and dexamethasone in relapsed multiple myeloma.

**Figure 2:**
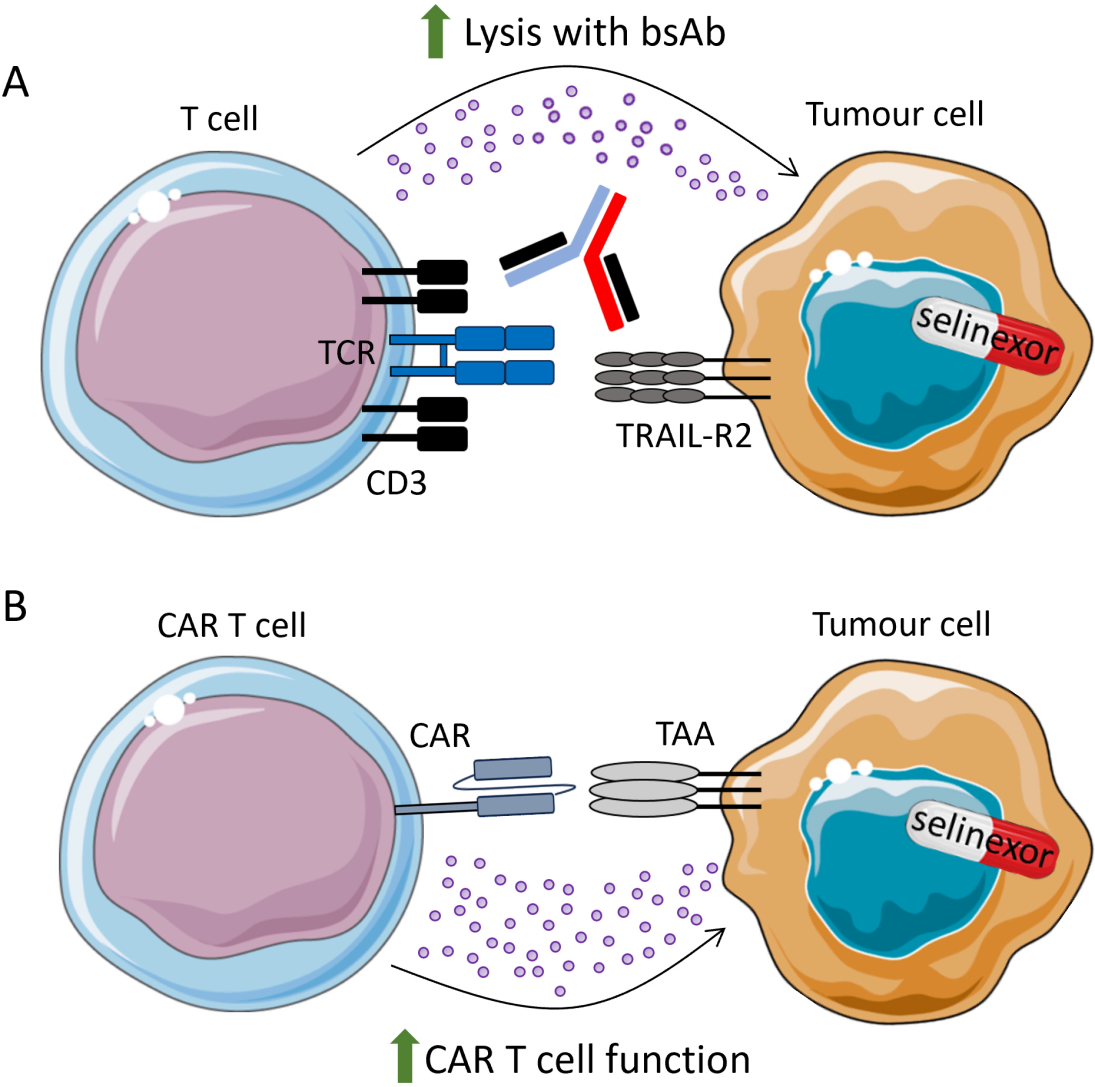
**Selinexor promotes bsAb and CAR-T cell function**. (**A**) Selinexor has been shown to sensitise TRAIL-R2^+^ breast cancer cells to CD3xTRAIL-R2 bispecific antibodies (bsAb); (**B**) in vitro and pre-clinical data have demonstrated that CAR-T cell function is enhanced when cancer cells are pre-treated with selinexor. Clinical trials are ongoing to combine selinexor treatment with adoptive transfer of anti-CD19 CAR-T and anti-BCMA CAR-T cells. TAA: tumour-associated antigen; TCR: T cell receptor; CAR: chimeric antigen receptor; BCMA: B cell maturation antigen; TRAIL: TNF-related apoptosis-inducing ligand. Parts of this figure were adapted from pictures provided by Servier Medical Art (https://smart.servier.com/) under a Creative Commons Attribution 4.0 Unported License (https://creativecommons.org/licenses/by/4.0/)

Bispecific antibodies, which target two separate proteins expressed on cancer cells and immune cells to aid their interaction, have recently been approved, including for the treatment of multiple myeloma and DLBCL, with several other targets under clinical investigation [89]. A bispecific targeting TRAIL-R2 and CD3 enabled the interaction of T cells with TRAIL-R2^+^ triple-negative breast cancer cells, an aggressive malignancy with poor prognosis [90]. Pre-treating breast cancer cell lines with selinexor enhanced TRAIL-R2xCD3 bispecific antibody-mediated growth inhibition of cancer cells and increased apoptosis when co-cultured with PBMC [90]. This indicates that XPO1 inhibition combined with T cell engagers may enhance anti-tumour immunity (Figure 2A) and this warrants further in vivo investigations.

As well as controlling NK cell function [62], NKG2A is also essential in regulating T cell activation [82, 91]. NKG2A is a late checkpoint receptor on T cells, expressed after multiple antigen stimulation rounds [92]. It is highly expressed on cytotoxic lymphocytes in the TME of liver, ovarian and bladder cancer, and the expression of NKG2A on tumour-infiltrating lymphocytes has been associated with poor prognosis in various cancer types [93–96]. In addition, Cazzetta et al. [97] (2021) identified an NKG2A^+^ Vδ2 T cell population with high anti-tumour activity in humans. This is in accordance with NKG2A being expressed on CD8^+^ T cells with a defined TCR clonality and committed T cell function [98, 99]. Recently, PD-1 and LAG-3 signaling were shown to limit T cell anti-tumour immune responses, and knockout of both genes induced NKG2A expression on tumour-specific T cells, illustrating the interplay between checkpoint receptor expression and highlighting the selective expression of NKG2A on tumour-specific T cells [100]. Interestingly, melanoma patients treated with anti-PD-1 (nivolumab) and anti-LAG-3 (relatlimab) in the RELATIVITY-047 clinical trial showed enhanced NKG2A expression on circulating, tumour-specific CD8^+^ T cells compared to pre-treatment. This indicates that targeting NKG2A in this setting may benefit anti-tumour immune responses [100]. Taken together, it will be interesting to determine whether selinexor-mediated inhibition of the HLA-E:NKG2A axis can promote the anti-tumour function of NKG2A^+^ CD8^+^ T cells with high tumour specificity.

Additionally, XPO1 inhibition can impair the production of the immunosuppressive cytokine IL-10, potentially preventing the inhibition of an ongoing anti-tumour immune response [73]. Interestingly, IL-10 has also been shown to enhance NKG2A expression on NK cells in liver cancer and high NKG2A expression is associated with poor prognosis [101]. As such, the inhibitory NKG2A:HLA-E axis may be modulated by XPO1 inhibition via two mechanisms within the TME: downregulating HLA-E on cancer cells and dampening IL-10 production.

## The effect of XPO1 inhibition on CAR-T cells

CAR-T cell therapy has revolutionized the treatment of certain haematological malignancies [102]. However, challenges remain with this nascent therapy, including optimizing apheresis and CAR-T manufacturing, preventing disease progression with bridging therapy while waiting for CAR-T cell production, cytokine-mediated side effects after CAR-T infusion, and relapse and lack of response to the CAR-T itself. Strategies are currently being evaluated to improve upon these challenges, amongst others. One area of investigation is CAR-T cell combination with small molecule inhibitors [103, 104]. Interestingly, Wang et al. [105] (2021) found that XPO1i can sensitise malignant B cells to lysis by anti-CD19 CAR-T cells (Figure 2B). This was only observed in pre-treatment experiments, as their function was not enhanced when selinexor was added to co-cultures of tumour cells + CAR-T cells [105]. These data are in accordance with the in vivo finding that sequential administration of selinexor to prime the TME and then anti-CD19 CAR-T cells promotes tumour regression compared to concurrent treatment [106]. Furthermore, recent data presented at ASH 2024 revealed that selinexor reduced M2 macrophages in a murine lymphoma model and improved CAR-T activity in vivo [107]. These studies have important implications for the optimal design of clinical trials which combine selinexor and CAR-T cells.

The first-in-human phase II clinical trial (NCT05322330) assessed anti-CD19 CAR-T cells in combination with selinexor as a bridging therapy in relapsed/refractory B cell non-Hodgkin lymphoma (NHL), with selinexor given at 40–60 mg per week for three weeks prior to CAR-T infusion. In early published results, the combination induced a response in four out of six patients [108]. Correlative data in these patients also showed that the use of selinexor resulted in a higher proportion of CD8^+^central memory T cell phenotypes while having no effect on CD4^+^ central memory T cell phenotypes. Additionally, in a recent study (NCT05201118), two patients with relapsed/refractory extramedullary multiple myeloma, an aggressive malignancy with poor survival, were treated with 40 mg selinexor as a bridging therapy, followed by lymphodepletion and administration of anti-B cell maturation antigen (BCMA) CAR-T cells [109]. Selinexor was administered again (40 mg or 60 mg weekly) to patients following CAR-T cell infusion as a maintenance therapy [109]. Both patients achieved deep, durable responses with regression of extramedullary disease for 10 months and 13 months at the time of data collection and although only a small pilot study, this highlights the potential benefit of using XPO1 inhibition to improve CAR-T cell activity. Although promising, both of these clinical trials have low patient numbers. Therefore, confirming these initial findings with a more extensive study and a longer follow-up period is critical.

A retrospective study on 7 RR multiple myeloma patients who received CAR-T and were treated with a selinexor-based regimen immediately prior to apheresis demonstrated an ORR of 100%, with all seven patients achieving excellent partial response or better [110]. Clinical trials assessing CAR-T cells in combination with selinexor are currently ongoing (Table 1), and the results of these trials will shed light on the potential of selinexor to potentiate CAR-T cell activity in patients.

Whether NKG2A expression on CAR-T cells played a role in these clinical trials was not investigated and the exact mechanisms for enhanced CAR-T cell activity of selinexor remain elusive. However, there is increased NKG2A expression on CD8^+^ T cells after multiple rounds of antigen stimulation [92], and consistent with this, elevated NKG2A expression was observed on CAR-T cells 28 days after infusion [111]. Increased NKG2A expression over time may reflect a highly activated, chronically stimulated CAR-T cell population with an exhausted phenotype [97]. As such, downregulation of HLA-E by selinexor may contribute to enhanced CAR-T cell activity, and this would be an interesting future area of investigation.

## The effect of XPO1 inhibition on B lymphocytes

Malignant B cells are sensitive to apoptosis with XPO1i [73] and selinexor is approved for the treatment of RR-DLBCL. Interestingly, however, healthy B cells are more resistant to apoptosis with XPO1 inhibition [73]. In terms of B cell function, the production of antibodies was only slightly delayed with selinexor in vivo (3x weekly dosing) compared to vehicle-treated mice*,* with minimal effect evident on antibody class switching [72]. Within the bone marrow, the site of early B cell development, selinexor reduced B cell proportions compared to vehicle, however, this recovered after two weeks, even with further doses of selinexor [72]. In mouse models of ovarian cancer, selinexor increased the proportion of splenic B cells, illustrating minimal impact on B cells within secondary lymphoid organs with XPO1 inhibition [88]. An initial report on infection risk with selinexor in multiple myeloma patients revealed a low risk of severe infection, potentially supporting evidence of a limited impact of selinexor on antibody-mediated immunity [112].

## Conclusions and future directions

The inhibition of XPO1 function has been shown in multiple preclinical studies to modulate immune cell function via several mechanisms (Table 2). Significantly for cancer treatment, XPO1 inhibition can promote a positive immune response against cancer via macrophage re-polarisation to the M1-like phenotype, modulation of T cell checkpoints, sensitisation of cancer cells to NK cell and T cell lysis, and inhibition of neutrophil extracellular traps. The mechanisms behind these immunogenic effects remain to be fully resolved. Early data from clinical trials combining selinexor with CAR-T cells are promising and are currently ongoing, future research should define the optimal sequencing of selinexor with CAR-T cells in patients. Furthermore, whether biomarkers can be utilized to personalize selinexor combination with immunotherapy, such as expression of HLA-E for NK cell-targeted therapies, remains to be determined and should be addressed in future studies.

**Table 2:** Effect of XPO1 inhibition on immune cell function

**Immune cell type**	**Effect on function**	**References**
Myeloid cells	Depletes lymphoma-associated macrophages.	[54]
	Reduces immunosuppressive effects of human MDSCs in a murine model of lymphoma.	[56]
	Macrophage polarisation from M2-like to M1-like within the TME.	[55]
	Increases macrophage abundance within the TME in murine models of pancreatic cancer.	[57]
	Increases MDSC infiltration in murine model of melanoma murine.	[58]
T cells	Reduces LAG-3, TIM-3 and PD-1 expression on tumour-infiltrating CD8^+^ T cells in murine models. Unaltered regulatory T cell infiltration in murine tumour models. Increased granzyme B and no induction of immune checkpoints in MM patient samples. At high concentrations impairs TCR signaling and T cell proliferation.	[58, 72]
	Pre-treatment of breast cancer cell lines enhances TRAIL-R2xCD3 bispecific antibody activity.	[90]
	Improves anti-CD19 CAR-T cell activity in vitro and in vivo.	[105, 106]
NK cells	Increases splenic NK cell abundance and does not alter numbers in the TME in murine models.	[58, 72]
	Increases NK cell-mediated lysis of tumour cells and ADCC due to downregulation of HLA-E on malignant B cells.	[61, 62]
Neutrophils	Neutropenia has been reported in patients.	[63]
	Impairs extracellular trap formation.	[64]
B cells	Initial reduction of B cells in the bone marrow in mice, which recovers during prolonged treatment.	[72]
	Minimal effect on antibody production or class switching.	[72]

MDSCs: myeloid-derived suppressor cells; TME: tumour microenvironment; LAG-3: lymphocyte activation gene-3; TIM-3: T-cell immunoglobulin and mucin-domain containing-3; PD-1: programmed cell death protein 1; TCR: T cell receptor; CAR: chimeric antigen receptor; NK: natural killer; ADCC: antibody-dependent cellular cytotoxicity; MM: multiple myeloma; XPO1: exportin-1; PD-1: programmed cell death protein 1; TRAIL: TNF-related apoptosis-inducing ligand

In patients, selinexor has been associated with neutropenia, gastrointestinal toxicity and fatigue; however, these can be mitigated by lower and less frequent selinexor dosing and by implementation of prophylactic measures [113]. The combination of immunotherapy with selinexor offers the potential for reduced dose and/or frequency of selinexor treatment to minimize adverse events. In addition, whether the next-generation XPO1i eltanexor possesses immunomodulatory properties remains to be determined. Future investigations into the effect of eltanexor on anti-cancer immunity are of high interest given the improved safety profile of eltanexor compared to selinexor [114], potentially due to its inability to penetrate the blood-brain barrier [115]. Increased knowledge of how XPO1i modulate the anti-cancer immune response will ultimately aid the design of future clinical studies to benefit more patients.

## Abbreviations

ADCC: antibody-dependent cellular cytotoxicity
BCMA: B cell maturation antigen
BTK: Bruton’s tyrosine kinase
CAR: chimeric antigen receptor
CLL: chronic lymphocytic leukaemia
CML: chronic myeloid leukaemia
CTLA-4: cytotoxic T-lymphocyte associated protein 4
DLBCL: diffuse large B cell lymphoma
GCB: germinal center B-cell
HDAC: histone deacetylase
LAG-3: lymphocyte activation gene-3
LMB: leptomycin B
mAb: monoclonal antibody
MDSCs: myeloid-derived suppressor cells
NHL: non-Hodgkin lymphoma
NK: natural killer
PBMC: peripheral blood mononuclear cells
PD-1: programmed cell death protein 1
PD-L1: programmed death ligand 1
RR: relapsed and refractory
TCR: T cell receptor
TIM-3: T-cell immunoglobulin and mucin-domain containing-3
TKI: tyrosine kinase inhibitor
TME: tumour microenvironment
TRAIL: TNF-related apoptosis-inducing ligand
Tregs: regulatory T cells
XPO1: exportin-1
XPO1i: exportin-1 inhibitor

## Declarations

### Copyright

© The Author(s) 2025.
